# Cardiac Arrest Following GoLytely Consumption: A Potential Trigger for Hypovolemic Shock

**DOI:** 10.7759/cureus.61486

**Published:** 2024-06-01

**Authors:** Eli A Zaher, Mohamed A Ebrahim, Parth Patel, Bibek Adhikari

**Affiliations:** 1 Internal Medicine, Ascension Saint Joseph Hospital - Chicago, Chicago, USA

**Keywords:** colonoscopy, cardiac arrest, laxatives, bowel preparation, golytely

## Abstract

This case report presents a rare but severe complication of polyethylene glycol (PEG) used for colonoscopic bowel preparation. A 71-year-old male developed cardiac arrest secondary to hypovolemic shock following consumption of GoLytely. Despite being hemodynamically stable prior to ingestion, the patient experienced extreme weakness, dizziness, and orthostatic hypotension post-consumption. Evaluation ruled out other causes of arrest. While serious complications from PEG are rare, this case underscores the importance of vigilance. Further investigation is warranted to elucidate the relationship between PEG use and cardiac events and to identify potential risk factors for adverse outcomes associated with bowel preparation regimens.

## Introduction

Bowel preparation for colonoscopy is considered one of the biggest barriers to adequate screening and diagnosis, with up to 25% of procedures having inadequate preparation. Polyethylene glycol (PEG) is one of the most commonly used laxatives for this purpose, with generally benign and minimal side effects apart from the expected diarrhea [1].

We report a case of cardiac arrest secondary to hypovolemic shock following PEG use. To our knowledge, this is only the second known case of cardiac arrest in the setting of PEG [2].

## Case presentation

A 71-year-old male with a history of diabetes and peripheral artery disease presented to the emergency department with complaints of chills and left foot pain. He was discharged a few days prior, following treatment of dry gangrene on the same foot with debridement and amputation of the left foot, along with endarterectomy and patch angioplasty of the ipsilateral lower extremity vessels. He reported that his post-operative wound was not healing properly, hence the decision to visit the emergency room.

Upon admission, his vital signs were consistent with sinus tachycardia to 110 bpm, temperature of 101°F, and normal blood pressure. Blood workup demonstrated chronic microcytic anemia (hemoglobin 8.5 g/dL, reference range 13.0-17.0) and leukocytosis (white blood cell count 13.0 k/mm³, reference range 4.0-11.0). Physical examination revealed a tender left lower extremity with wounds consistent with a recent left foot amputation. No tenderness or visible discharges were observed. The left lower extremity was warm with palpable pulses. 

Our patient was thus admitted for management of sepsis secondary to suspected post-operative infection, with intravenous fluid resuscitation and intravenous antibiotics. Both wound and blood cultures were collected prior to the initiation of antibiotics. 

Over the course of his hospital stay, the patient was afebrile and his leukocytosis resolved. His blood pressure was likewise within normal limits, without further episodes of tachycardia. The post-operative wound and blood cultures were both negative. 

**Figure 1:**
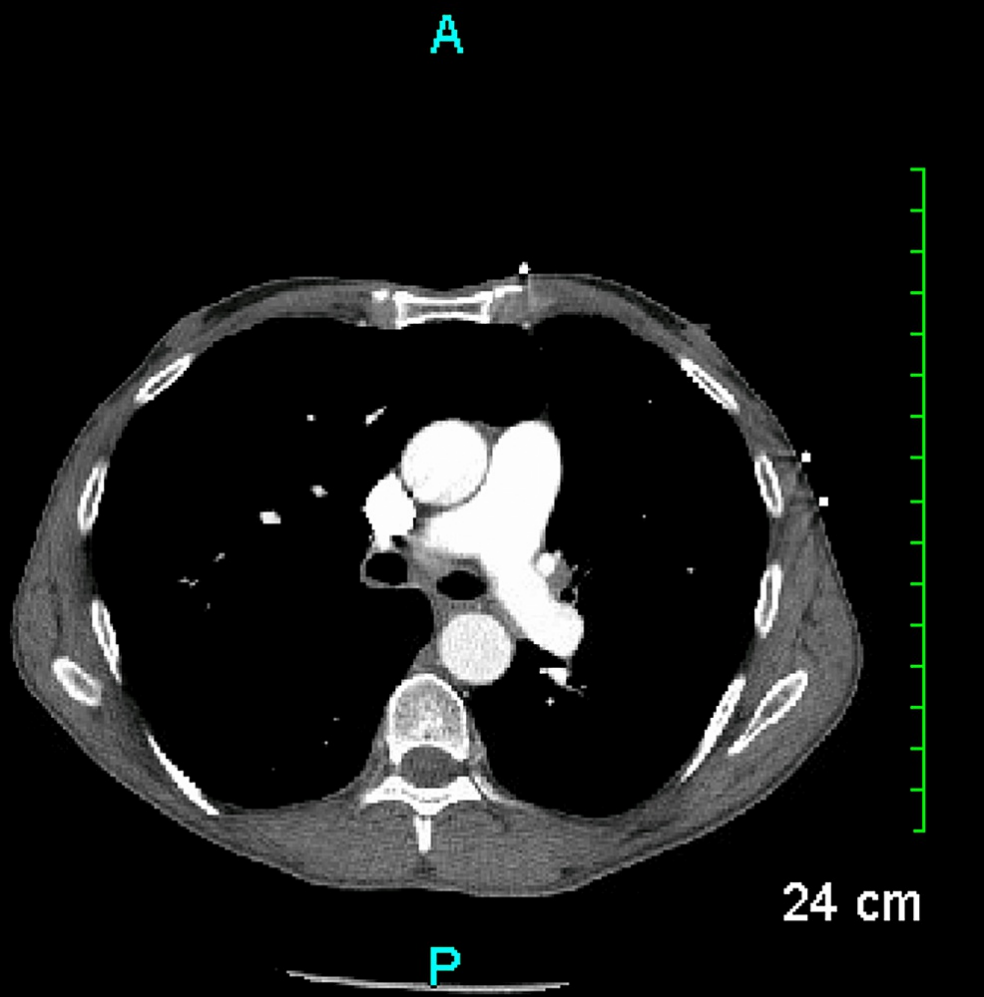
CT angiography negative for pulmonary embolism

His hemoglobin level remained stable at around 8.5 g/dL throughout his stay, without signs of bleeding. Given he never had colorectal screening and the concurrent anemia, the patient opted to proceed with an index colonoscopy prior to discharge. He was given GoLytely (polyethylene glycol 3350 and electrolytes) for bowel preparation on the day prior to the procedure. The patient reported multiple and extensive watery bowel movements beginning after he started drinking the solution. The following morning, the patient had completely drunk the GoLytely solution but reported feeling very weak and dizzy from the frequent diarrheas overnight. His vital signs were positive for orthostatic blood pressure. Laboratory workup from the same morning was consistent with creatinine elevation to 1.8 mg/dL from the previous day's level of 1.2 mg/dL (reference range 0.7-1.3) and non-anion gap metabolic acidosis (bicarbonate 18 mmol/L, reference range 22-32) without electrolyte abnormalities. His colonoscopy was thus canceled, and he was initiated on intravenous fluid resuscitation. Shortly thereafter, the patient was found unresponsive in his room, without palpable pulses. Cardiopulmonary resuscitation was begun, with a return of spontaneous circulation achieved in four minutes. Post-cardiac arrest workup was negative for distributive, cardiogenic, and obstructive shock causes, raising the suspicion of severe hypovolemic shock secondary to diarrhea (Figure 1).

## Discussion

As other medications are used to prepare the bowels for medical procedures, GoLytely operates by inducing diarrhea, which effectively cleanses the bowel without being absorbed into the bloodstream. This means that dosage adjustments are unnecessary for patients with liver, kidney, or other health issues. The package inserts labels common side effects that may include nausea, abdominal bloating, and a feeling of fullness, affecting about half of those who use GoLytely. Less frequent adverse reactions include abdominal cramps, vomiting, and anal irritation [3]. While serious complications such as pulmonary aspiration, cardiac arrhythmias, seizures, colitis, and electrolyte imbalances have been reported, they are rare [4]. Despite the potential for temporary changes in electrolyte levels, GoLytely appears to be safe for patients with fluid and electrolyte imbalances due to its balanced osmolarity.

Cardiac arrest can stem from either cardiac or noncardiac origins. Cardiac triggers encompass conditions like acute or chronic heart failure, myocarditis, aortic dissection, hypertrophic cardiomyopathy, aortic valve disease, use of cardiotoxic medications, and complete heart block. Noncardiac factors include pulmonary embolism, intracranial hemorrhage, drug overdose, and trauma [5]. After our patient completely consumed all of the GoLytely solution, he complained of extreme weakness and dizziness due to frequent diarrhea episodes overnight. His vital signs indicated orthostatic hypotension. The patient was hemodynamically stable preceding the administration of the GoLytely solution, with vital signs such as blood pressure, heart rate, and respiratory rate within normal ranges. Furthermore, postcardiac arrest evaluations, including echocardiography and electrocardiograms, ruled out myocardial infarction, cardiomyopathy, and arrhythmias as potential causes. Additionally, there were no signs suggestive of pulmonary embolism. After excluding other causes of cardiac arrest, GoLytely was considered a potential culprit.

Cardiac issues associated with PEG-electrolyte solutions like GoLytely, Colyte®, and Nulytely® are rare but documented. A study comparing sodium phosphate (NaP) and PEG-electrolyte solutions found no significant adverse effects with either, but more patients using PEG-electrolyte solutions experienced drops in systolic blood pressure [6]. Additionally, reports show instances of serious adverse events, including cardiac arrest, linked to colon cleansing preparations. A review of the FDA adverse event reporting system (FAERS) shows numerous adverse events relating to PEG-electrolyte solutions, with some cases involving serious cardiac abnormalities [7]. Despite its constraints, the FAERS lacks medical validation and permits reports from various sources, including healthcare providers and consumers. It does not restrict the number of times an event can be reported and is not comprehensive, as participation is voluntary. While the FDA database has limitations, the FAERS database remains a valuable resource for uncovering potential serious adverse effects associated with GoLytely, as it offers valuable insights into potential serious effects of these solutions.

## Conclusions

While GoLytely appears generally safe for most patients, clinicians should remain vigilant regarding its infrequent, yet severe complications. Although this case report and analysis of adverse reports hint at a possible association between GoLytely and cardiac arrest, further investigation is necessary to address several key points: the causal connection between GoLytely and cardiac arrest; potential risk factors for life-threatening adverse events linked to GoLytely; and serious adverse effects associated with all PEG products, with or without electrolytes.
